# Tuberculosis and Its Prophylaxis*Read at meeting Central Texas Medical Society.

**Published:** 1906-10

**Authors:** O. I. Halbert

**Affiliations:** Waco, Texas


THE
### TEXAS MEDICAL JOURNAL.
Established July, 1885.

F. E. DANIEL, M. D., - - - - - Editor, Publisher and Proprietor.

PUBLISHED MONTHLY.—SUBSCRIPTION $1.00 A YEAR.

VOL. XXII. AUSTIN, OCTOBER, 1906. No. 3.

The publisher is not responsible for the views of contributors.

For Texas Medical Journal.

### Tuberculosis and Its Prophylaxis.*

BY O. I. HALBERT, M. D., WACO, TEXAS.

I do not expect to offer to you anything new or startling, but simply "stir up your pure minds by way of remembrance."

"It is in the power of man to cause all parasitic diseases to disappear from the world."—Pasteur.

"Prevention is better than cure and far cheaper," said the great philosopher, John Lock.

Tuberculosis is an infectious, contagious disease. It has been known and dreaded as far back as the memory of man runs, for it has decimated the human family in every clime, and at all altitudes where'er they are found. The mortality reports of Germany for 1894 show that while 116,705 died of diphtheria, croup, whooping-cough, measles, scarlet fever and typhoid fever, 123,904 died of tuberculosis alone. It is estimated that 150,000 die of tuberculosis annually in the United States, and 5000 of which die in Texas alone. This is over 400 deaths per day in the United States. Is the estimate that one-seventh of all deaths are due to the great white plague too high?

But this is not all, from a politico economic view, it is still more dreadful. These other infectious and contagious diseases, such as diphtheria, croup, measles, scarlet fever, etc., gather their victims from the children mostly. Tuberculosis gathers more than 75 per cent of its victims from bread winners, between the ages of 15 and 75 years. Add to this fact another: Consumption runs a number

*Read at meeting Central Texas Medical Society.

of years, upon an average of about three, before it proves fatal. If you rate the time lost from invalidism, the expense of doctoring, or nursing, of medicine, of food, of clothing, and of burial, you have some idea of the costliness of this preventable scourge. Prof. Carnet has estimated from authentic statistics that tuberculosis costs the Prussian States 86,000,000 marks, or $20,468,000, per year; an increase tax of a little less than 74 cents per capita. Surely "Prevention is better than cure, and far cheaper."

Pulmonary tuberculosis is the form most frequently met, nearly 93 per cent of all cases for one year in the German Empire; yet it attacks every other tissue in the body, from the meninges of the brain to tuberculosis of the joints, from lupus of the skin to tubercular peritonitis, and in every tissue the tubercle bacillus is the constant and ever-to-be-feared factor of the disease. While tuberculosis was described by the classic old Greek physician, Hypocrates, nearly five hundred years before the birth of our Savior, and has been studied and written about ever since, it waited until the nineteenth century to receive the greatest revelation yet. In 1882 the accomplished German physician and bacteriologist, Robert Koch of Berlin, published his discovery that all forms of tuberculosis are due to the tubercle bacillus. This was proven, not alone by the presence of this distinct bacillus in every form of tuberculosis, but he inoculated animals with tubercular pus, and produced tuberculosis. He went still further, and isolated, and grew on aseptic blood serum, colonies of these peculiar bacilli, and injected these into guinea pigs and rabbits, and every one of these were infected with tuberculosis. This is perhaps the most important discovery ever made in medicine, because it proved the bacillary origin of this, the greatest destroyer of our races, and opened the enemy to the fire of scientific medicine. These little bacilli, which can only be seen with an Oil-Emersion Abbe Illuminator and an open iris diaphragm, appear as slender rods, varying in size, but on the average equalling one-fourth or one-half the diameter of a red blood corpuscle. To make it more thinkable, it would take 6350 to 8466 put end to end to extend an inch. These little dynamos of destruction and death are thrown off from animals and human beings only in their secretions and excretions, and the flesh of these infected animals may carry these germs to the ones who eat it. The only way of taking the infection is through the respiratory and alimentary tracts, with the rare exception of taking through an absorption of the skin or mucous membrane, and still rarer of inheritance. It can be inoculated anywhere in the body. The heredity of tuberculosis has been hotly discussed and many scientific experiments have been reported for and against it. I believe I can best give my conclusions in the language of Prof. G. Carnet, of Berlin, whose excellent book should be in the library of every physician. He says: "The assumption of a seminal transmission of the tubercle bacillus (a hereditary transmission from the paternal side) lacks all foundation. However, the maternal transmission is undoubtedly possible but up to the present time the extremely small number of recorded cases, in spite of the fact that for many years the attention of numerous pathologists has been constantly directed to this point, together with the results of animal experiments, as well as clinical, pathological and statistical data, make it appear that such transmission is extremely rare and dependent on special conditions, such that one should be exceedingly skeptical in believing any reports of inherited tuberculosis." There are only twenty authenticated cases so far reported. Of these, one lived only three weeks, another eight days and the other eighteen were premature labor and still-born. I doubt not that many more have existed, but have either not been recognized or, if recognized, not reported. While very few inherit the disease itself, a great number inherit environments and become infected soon after birth. Many instances may be cited to prove this to be the way in which the offsprings inherit tuberculosis. Ipstein observed, at the Foundling Asylum, at Prague, that children of tubercular mothers nursed by healthy wet nurses flourished, whereas those nursed by their sick mothers soon died of tuberculosis. In four years he failed to discover a case of tuberculosis in nursing infants, at the Foundling Asylum. At the Orphan Asylum of Nurnberg, with a capacity of one hundred children, only one single case of tuberculosis was discovered in eight years, although there were many of decided tubercular family history. Dr. Berheim had in his pratice three cases of twins of tubercular mothers. He sent one of each pair to the country and had them nursed and cared for by healthy wet nurses, these three grew hale and hearty, while the three left with their mothers, although suckled by healthy wet nurses, all soon died. Such examples, and there are a great many, show clearly where the responsibility lies, since we have so much tuberculosis in our asylums, orphanages, and homes of our people. While we do not inherit the disease, it is undeniable that the children from consumptive parents, especially consumptive mothers, or chronic alcoholics, or any other serious constitutional disease, have a weakness, or somatic inferiority, and are, therefore, more prone to tuberculosis, as well as any and all other prevalent diseases.

The "habitus phthysicus," characterized by a narrow chest, with a long vertical diameter, and a long thin neck, is the most expressive sign of hereditary predisposition. Nearly all of the human family are exposed to the contagion of tuberculosis is proven by the fact that one-seventh die of this disease, and many dead of other diseases are found infected with the tubercle bacillus, or to have been infected. This being true, shows that some are more susceptible than others. True though this be, the fact that athletes and others, the very embodiment of physical strength, as well as prize-fat cattle, take tuberculosis and die with it, proves that hereditary predisposition is not so unfortunate as hereditary exposure.

We have not advanced much in the therapeutic treatment of consumption. The drugs that will destroy the tubercle bacillus in the patient will also destroy the patient. We confidently hope that the time will soon come when treatment shall prevail. The Rockefeller and Carnegie Institutions for scientific research, helped by all other scientific work now in progress all over the civilized world, are bound to prevail, and a cure for tuberculosis, I predict, will soon be proclaimed. While this is in the near future, the proper prophylaxis is now established, and we can and must prevent it. The first point I want to emphasize is that tuberculosis is not inherited, except in the rarest cases, but is caught, just as smallpox, measles, whooping-cough, and all other contagious and infectious diseases are. The second point, the materies morbi is thoroughly known and understood; its habitat, its modes of conveyance, its necessary pabulum, its incompatibilities and affinities, are as well known and as thoroughly understood as any other scientific facts. We know that the tubercle bacillus is gotten either from the secretions and excretions of infected persons and animals or from the milk and meat of tuberculous animals. We divide prophylaxis into State and municipal, and individual.

As this disease costs the States and cities such enormous amounts in dollars and cents, we will look at it from a politico economic standpoint. We have before stated that consumption costs the Prussian States $20,468,000 per year, an annual increased tax of $3.57 for each family of five members; besides this direct expense, it costs an inestimable amount in pauperizing large families, in depriving them of their natural support in the death of their parents. Every State, county, and city should have sufficient amounts set aside in their budget of expense, each and every year, to fight the spread of this scourge. The State Health Department should take charge of tuberculosis as they do yellow fever, smallpox and other quarantinable diseases. Sanatoria should be provided and kept at the expense of the State for all patients suffering with this disease and who are not able to be properly cared for at home. All railroads should be properly, systematically disinfected. Acting through county officers, and they through boards of health in municipalities, all slaughter houses and dairy herds should be properly inspected, and no tuberculous meat and milk should be sold. All schools—State, county and municipal—should be regularly and efficiently inspected by competent persons, and all cases, be they teacher, pupil or janitor, with suspicious symptoms of tuberculosis, should be either segregated until they are either restored to health or diagnosis, is positive, and when positive, they should be dismissed from school. No tuberculous person should be allowed in any school. All towns and cities should pass stringent laws against spitting on floors, sidewalks, open grates, and other such places; and any violations of such laws should be punished severely. All public resorts, such as schools, hotels, railroad stations, public libraries and other places, where people congregate should have plenty of cuspidors properly filled with antiseptics and properly cared for, and stringent laws enforcing these measures should be passed.

All consumptives, their names, residences, occupations, etc., should be properly reported to health authorities, and they should be regularly visited by the health officers, instructed by them, and forced to carry out measures that would prevent the spread of the disease. After the death of every consumptive the house in which he lived should be properly disinfected. Every house vacated by a consumptive should be disinfected. Every county and every city of 5000 or more inhabitants should have a competent and well-paid bacteriologist, whose duty should be to examine the sputum of all suspicious cases reported. Every up to date physician should have a clinical laboratory and be able to examine sputum and detect the tubercle bacillus, just as he would casts and pus in the urine. All buildings in course of construction, both public and private, should be supervised by a competent committee, so as to insure proper amounts of sunshine and ventilation in all parts of the building.

Individual prophylaxis. Remember that we catch consumption either from the meat or milk of tuberculous animals or from the secretions or excretions of tuberculous human beings, with only very rare exceptions; and that the sputum is by far the most frequent conveyor of the infection, drying and being breathed into throats and lungs, and being conveyed into stomachs and intestines with food. It is estimated that a consumptive, coughing on an average of once every hour, will throw off seven billion of these bacilli each twenty-four hours. These germs live from a few hours to six months, after being expectorated, being easily killed by sunlight and meteorological influences. They live longest in damp, dark places, and have been found alive three years after being expectorated. As physicians, we should forbid tuberculous mothers nursing their offsprings. Delicate, frail children should be reared in the country; if not practicable, should be kept in well-ventilated and well-sunned apartments, and should be kept out of doors as much as practicable, should have their skins, early in life, hardened by sponging all over the body with cold salt water. They should be fed on fats and plain foods and avoid sweets and other trash, should be made to wear loose fitting garments, to avoid awkward, cramped attitudes. All obstructions to air passages should be removed, such as adenoids, enlarged tonsils, and deviations in nostrils corrected. They should not be pushed in their studies, should not be allowed to associate with consumptives, except out in the open air, and not allowed to select an indoor or sedentary life, but, if possible, a life on a ranch or farm, and don't let such select a frail, delicate partner for life's journey.

In conclusion, we, as physicians, should explain to our tuberculous patients how dangerous they are to themselves and their loved ones, and even their friends and acquaintances, if they do not strictly follow the law of prophylaxis; and how perfectly harmless they are if they do. To you, gentlemen, it is only necessary to say that, realizing the sputum to be the greatest source of infection, we should go into the most detailed directions, how to absolutely prevent any of it from drying up and infecting the air of the apartments or the foods to be eaten. The urine, feces and saliva are not so dangerous, but should be thoroughly disinfected or destroyed.

Remember that Pasteur has said: "It is in the power of man to cause all parasitic diseases to disappear from the world." Tuberculosis is a parasitic disease. *Shall we not stamp it out?*

